# Gastric adenocarcinoma with submucosal invasion occurs in a sporadic fundic gland polyp of a *Helicobacter pylori*-uninfected patient on proton pump inhibitor

**DOI:** 10.1007/s12328-025-02167-2

**Published:** 2025-06-27

**Authors:** Haruki Kanai, Kengo Kasuga, Sakuya Katakai, Shingo Ishihara, Yoshiyasu Takayama, Xing Hua Ma, Hiroshi Kosugi, Takashige Masuo, Yoji Takeuchi, Toshio Uraoka

**Affiliations:** 1 Department of Gastroenterology, Isesaki Municipal Hospital, 12‐1 tsunatorihon‐machi, Isesaki, 372‐0817 Japan; 2 Department of Pathology, Isesaki Municipal Hospital, Isesaki, Japan; 3 Kosugi Internal Medicine Clinic, Isesaki, Japan; 4 Department of Gastroenterology and Hepatology, Gunma University Graduate School of Medicine, Maebashi, Japan

**Keywords:** Stomach neoplasms, Fundic gland polyp, *Helicobacter pylori* negative

## Abstract

Gastric fundic gland polyps (FGPs) are common benign lesions, typically found in *Helicobacter pylori* (HP)-uninfected stomachs. While syndromic FGPs resulting from genetic diseases may exhibit dysplasia, sporadic FGPs rarely develop into adenocarcinomas. Here, we present the first case of invasive gastric adenocarcinoma that occurred in a sporadic FGP in an HP-uninfected patient. A 77-year-old man on proton pump inhibitor (PPI) therapy for 6 years developed an enlarged reddish FGP. The absence of atrophic changes in the entire gastric mucosa was confirmed endoscopically and multiple HP tests were negative. After PPI discontinuation, the lesion initially reduced in size from 25 to 15 mm in 1 month; however, malignancy was suspected because of rapid enlargement and persistent discoloration. En bloc resection was performed by endoscopic mucosal resection. Histological examination confirmed FGP and submucosal adenocarcinoma with a depth of approximately 700 μm, negative for lymphovascular invasion, and negative resection margins. A subsequent distal gastrectomy confirmed the absence of residual disease or lymph node metastases. The unusual endoscopic findings of rapid enlargement and reddish tone pushed us to perform endoscopic treatment, which resulted in a correct diagnosis and appropriate treatment.

## Introduction

Gastric fundic gland polyps (FGPs) are common benign polyps found in *Helicobacter pylori* (HP)-uninfected stomachs [1]. They consist of cystically dilated fundic glands beneath the normal gastric foveolar epithelium. They may occur sporadically or as part of several polyposis syndromes, including familial adenomatous polyposis (FAP) and gastric adenocarcinoma, and proximal polyposis of the stomach (GAPPS) [2]. Syndromic FGPs often involve epithelial dysplasia, whereas dysplastic lesions in sporadic FGPs are rare and no cases of invasive carcinoma have been reported. Herein, we report the first case of invasive gastric adenocarcinoma that occurred in a sporadic FGP in an HP-uninfected stomach.

## Case report

**Fig. 1:**
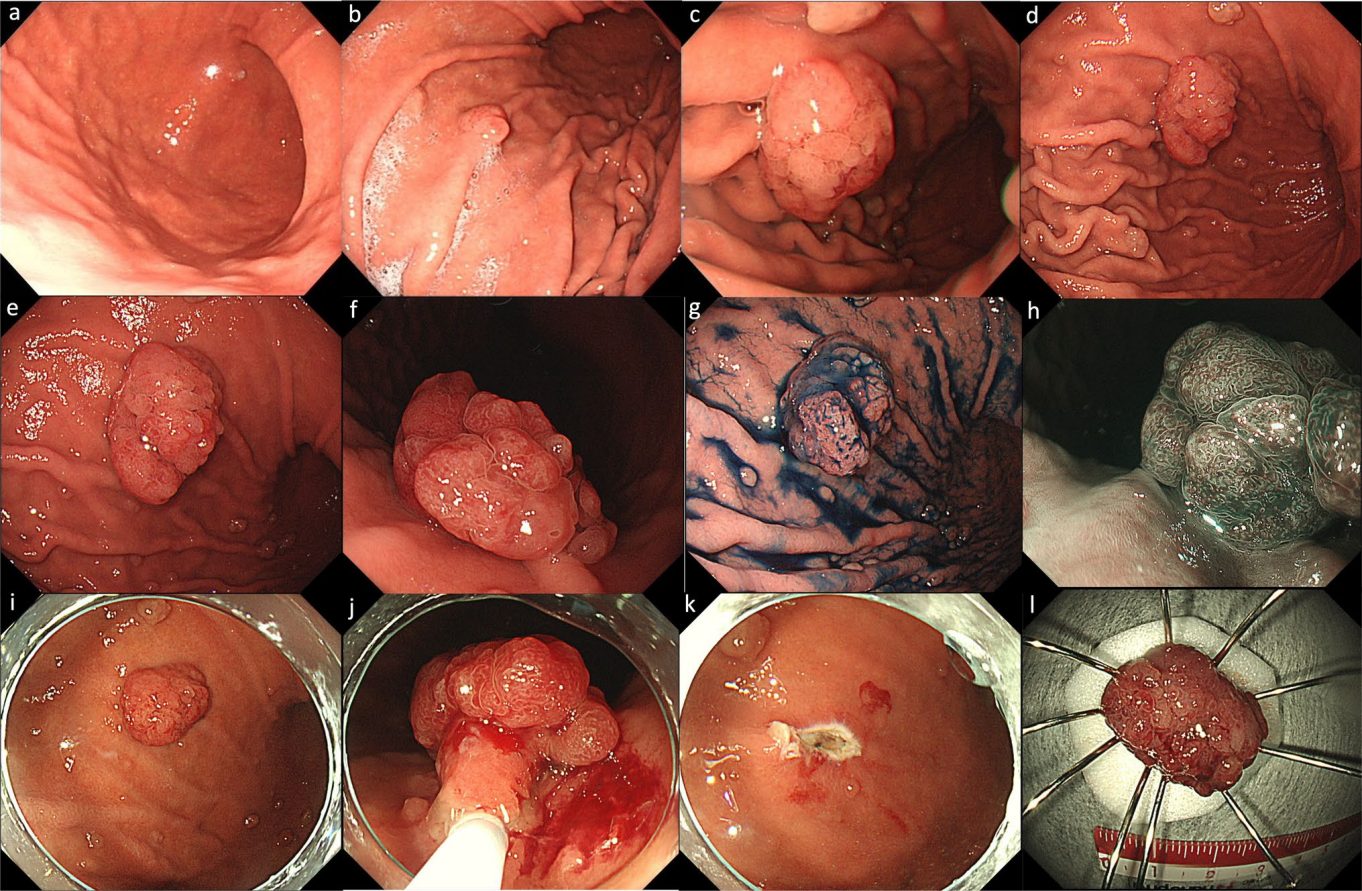
Endoscopic findings. **a** 6 years before referral at family doctor showed only several fundic gland polyps (FGPs). **b** 4 years before at family doctor showed a gradual increase in the number and lesion size of FGPs. **c** Just before referral at family doctor showed an enlarged reddish polypoid lesion. **d** White light imaging revealed a partially reddish polypoid lesion 25 mm in size surrounded by isochromatic FGPs in the greater curvature of the gastric body. **e** Close observation of the former. **f** In retroflex view, the posterior wall side was reddish and uneven on the surface. **g** With chromoendoscopy with indigo carmine. **h** Magnifying endoscopy with narrow band imaging of the reddish area. **i** One month later, the reduction in the previously identified polypoid lesion and the surrounding FGPs. **j** During endoscopic mucosal resection procedure. **k** Post-resection wound surface. **l** Resected tumor specimen

**Fig. 2:**
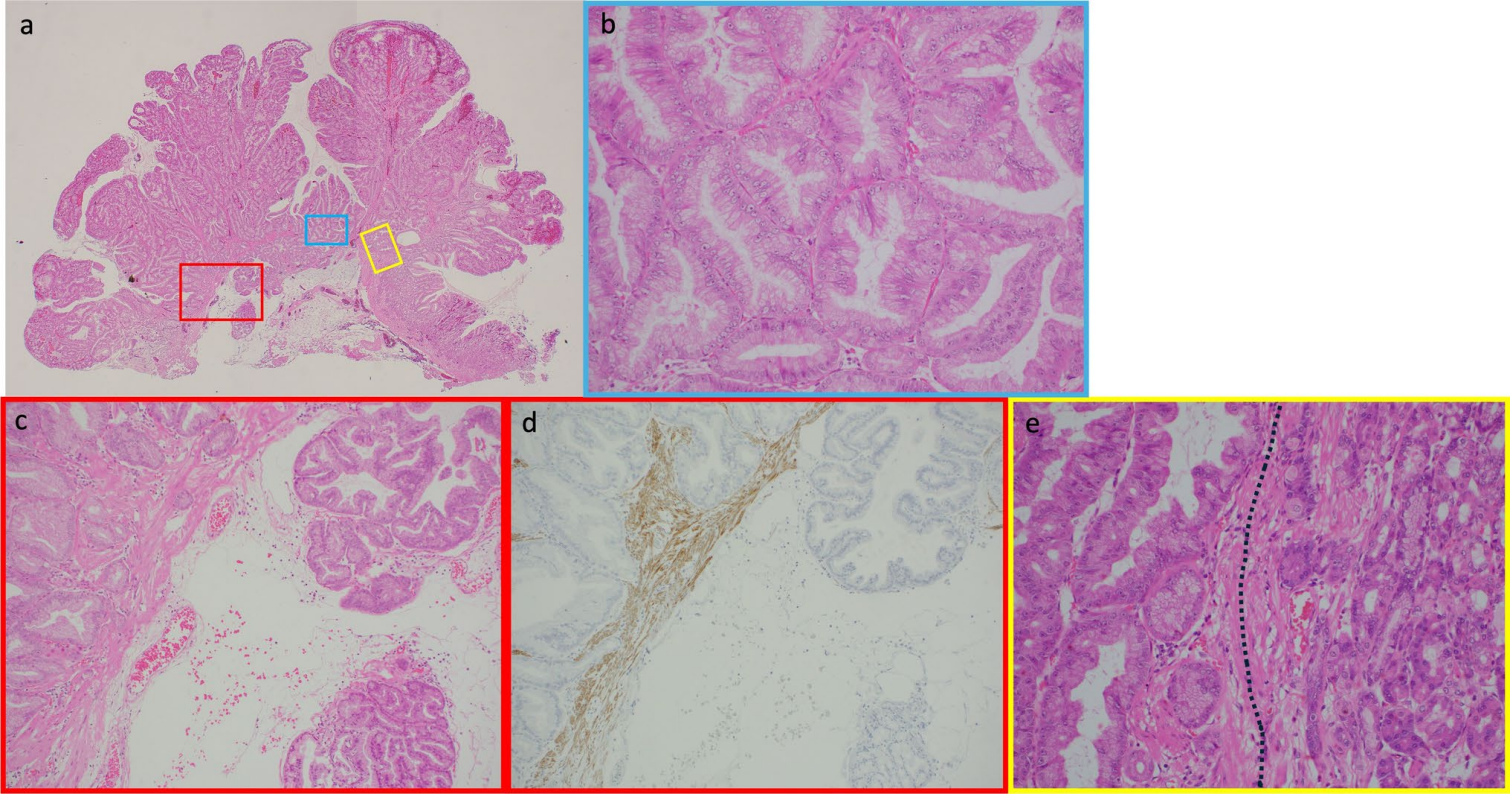
Histopathological findings of the EMR specimen **a** Semi-macro images of the entire lesion in the resected specimen indicating the lesion to be a submucosal adenocarcinoma and no tumor involvement of the resection margins. **b** High magnification of a cancerous area. Tumor cells formed branching tumor glands and exhibited round, enlarged, hyperchromatic nuclei, with a diagnosis of well-differentiated adenocarcinoma (magnification × 200). **c** Higher magnification of submucosal invasion (magnification × 100). **d** Desmin stain indicated that a muscularis mucosa and adenocarcinoma had infiltrated the submucosal layer (magnification × 100). **e** The boundary between adenocarcinoma and FGP. The area to the left of the dashed line is adenocarcinoma, and the area to the right is FGP (magnification × 200)

**Fig. 3:**
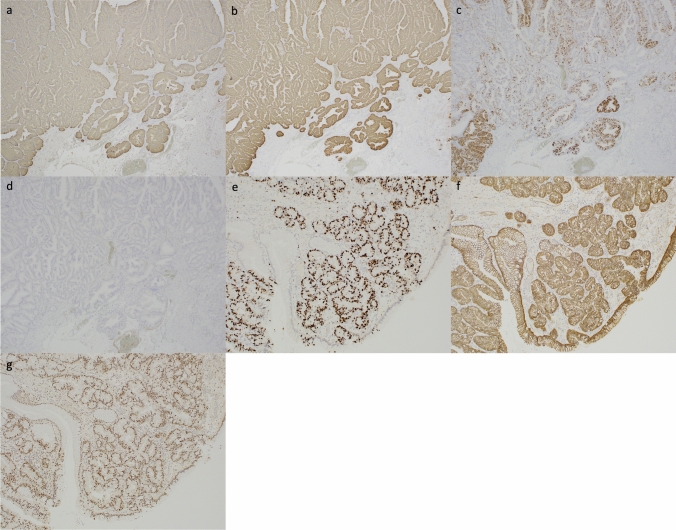
Immunohistochemical findings of the adenocarcinoma component (magnification × 100). **a** Positive expression of MUC5AC. **b** Positive expression of MUC6. **c** Partial positive expression for MUC2 expression of MUC2. **d** Negative expression of CD10 **e** Ki-67 was diffusely positive. **f** β-Catenin accumulation was observed in the cell membrane. **g** Overexpression of p53 was observed

A 77-year-old man underwent esophagogastroduodenoscopy (EGD) for a gastric cancer checkup 6 years earlier by a family doctor, which revealed reflux esophagitis and several FGPs (Fig. 1a). Subsequently, a proton pump inhibitor (PPI) (esomeprazole; 20 mg once daily) was continuously administered, and an annual EGD was performed, which showed a gradual increase in the number and lesion size of the FGPs (Fig. 1b). At the last examination, an enlarged reddish polypoid lesion was detected (Fig. 1c), which was histologically diagnosed as a gastric adenoma on a biopsy sample. The patient was referred to our institution for further examination and treatment. Physical examination revealed no remarkable physical findings. His medical history included the presence of appendicitis and ulcerative proctitis. He was a former smoker with a 30-year history of smoking 20 cigarettes per day. He had no history of habitual alcohol consumption. Laboratory data were within normal limits, including carcinoembryonic antigen and carbohydrate antigen 19-9 levels. Colonic polyps were not detected on colonoscopy and he had not received HP eradication therapy. He had no family history of colon or gastric cancers. Laboratory data were within the normal range, and investigations for HP demonstrated that the serum level of anti-Hp antibody was negative, and the urease breath test was within the normal range. EGD revealed no atrophic changes in the gastric mucosa. Duodenal polyps were not detected. There was a partially reddish polypoid lesion, 25 mm in size, surrounded by many isochromatic FGPs in the greater curvature of the gastric body (Fig. 1d); close observation of the former revealed that the anterior wall side was isochromatic and smooth on the surface (Fig. 1e), and the posterior wall side was reddish and uneven on the surface (Fig. 1f). With chromoendoscopy with indigo carmine, the boundary of the lesion was clearly visible (Fig. 1 g). Magnifying endoscopy with narrow-band imaging of the posterior wall side of the lesion revealed irregularly arranged dot-like crypt openings, densely arranged vessels, and no irregular microvessels (Fig. 1h). A biopsy of the reddish area was performed. Histological examination of the biopsy specimens revealed that it was difficult to differentiate between inflammation and tumors. After EGD, the PPI was discontinued because of a suspected increase in FGPs caused by the PPI and switched to a histamine-2 receptor blocker (famotidine 40 mg/day). One month later, EGD was performed. Examination revealed a reduction in the previously identified polypoid lesion and surrounding FGPs (Fig. 1i). Follow-up observation was considered; however, due to findings such as redness and rapid growth, malignancy was suspected, and endoscopic mucosal resection (EMR) was performed (Fig. 1j, k, l). Histological examination revealed that the lesion was a well-differentiated adenocarcinoma occurring within the FGP and infiltrated into the submucosal layer (Fig. 2a, b, c, d). Neither atrophy nor intestinal metaplasia was detected. Common features of FGPs, such as cystically dilated fundic glands, were observed near cancerous areas. In addition, foveolar cell proliferation and protruding parietal cells, which are characteristic of PPI-associated FGP, were not observed (Fig. 2e). Some of the atypical cells exceeded the desmin stain-positive muscularis mucosa and infiltrated into the submucosal layer, with a depth of approximately 700 μm, negative for lymphovascular invasion (D2-40, Elastica Van Gieson), and negative resection margins. Immunohistochemistry revealed that the neoplastic cells were positive for MUC5AC (Fig. 3a) and MUC6 (Fig. 3b); partially positive for MUC2 (Fig. 3c); and negative for CD10 (Fig. 3d), pepsinogen I, and H + /K + ATPase. Ki-67 was diffusely positive in the cancerous area (Fig. 3e), and β-catenin accumulation was observed only at the cell membrane and there was no accumulation in the nucleus (Fig. 3f). Also, overexpression of p53 was observed (Fig. 3g). One month later, distal gastrectomy with lymph node dissection was performed. The pathological diagnosis of the resected specimen showed no local residual disease or lymph node metastasis. As with the EMR specimens, no gastric mucosal atrophy or intestinal metaplasia was observed in the surgical specimens.

## Discussion

FGP is a benign gastric polyp, often found in the stomach without atrophic changes, and occurs both syndromically and sporadically, with background disease in the former, including FAP and GAPPS [3, 4]. Sporadic FGP is known to be associated with PPI use, and its frequency has increased with the recent increase in PPI use [5, 6]. Its frequency varies among reports; however, in the general population, it ranges from 1.8% to 5.9%, with a predilection for middle-aged women [1, 7]. Syndromic FGP is often associated with epithelial dysplasia; however, sporadic FGP with high-grade dysplasia or adenocarcinoma is extremely rare [8]. Recent studies have suggested that long-term gastric acid suppression is associated with carcinogenesis, but these studies have been limited to gastric cancer after HP eradication [9, 10]. It is unclear whether HP-uninfected gastric cancers are associated with long-term gastric acid suppression, perhaps because HP-uninfected gastric cancers are a small proportion of all gastric cancers. Sporadic FGPs show a high probability of activating mutations in CTNNB1 (encoding β-catenin) [11], while syndromic FGPs are caused by inherited germline mutations in the adenomatous polyposis coli (APC) gene with somatic second hit mutations [12]. Non-dysplastic sporadic FGPs only have activating mutations in CTNNB1, whereas dysplastic FGPs, both sporadic and syndromic, have been reported to have truncating mutations in the APC gene [13]. In the present case, the absence of colorectal cancer in the patient's family history and the absence of adenomatous polyposis on colonoscopy suggested that the lesion was a gastric adenocarcinoma occurring in sporadic FGP that had increased with oral PPIs. However, our institution was not equipped to carry out genetic analysis, and we could not perform it.

To the best of our knowledge, only six cases of sporadic FGP with high-grade dysplasia or adenocarcinoma have been reported, and none of them were invasive carcinomas [14–18]; five of the six cases were without HP infection, and the remaining one occurred after HP eradication. Two of the five cases in which PPI use was mentioned were on PPIs for long periods (5 and 14 years). Of the six cases, four had a reddish tone. The remaining two cases did not mention the color tone. In this case, there was no evidence of HP infection, the patient had been on PPI for 6 years, and the lesion was observed to be reddish; although FGP is essentially isochromatic, the reddish color of FGP suggested the possibility of adenocarcinoma. Furthermore, FGP enlarged by PPI regresses in approximately 2 months [19]. In this case, the lesion also reduced in size from 25 to 15 mm after discontinuation of PPI for 1 month. Therefore, even if the PPI withdrawal resulted in a reduction of the lesion, malignancy cannot be ruled out. Furthermore, in pathological findings, foveolar cell proliferation and parietal cell protrusion, which are typical findings in PPI-associated FGPs [20, 21], were not prominent, suggesting the possibility of regression due to PPI withdrawal.

In immunochemical staining, Jalving et al. [22] and Nawata et al. [18] reported that nuclear staining for β-catenin was observed in sporadic FGP dysplasia and that activation of the Wnt–APC-β-catenin pathway may be involved in the development of dysplasia, but in this case, no nuclear staining for β-catenin was observed. There are also reports that no nuclear staining for β-catenin was observed in sporadic FGP dysplasia, suggesting that pathways other than the APC-β-catenin may be involved in the development of FGP high-grade dysplasia or adenocarcinoma [14, 16, 17]. The overexpression of p53, which is not typical of FGP dysplasia [23], was observed in this case. The patient had a history of ulcerative proctitis, which is known to be associated with p53 mutations at an early stage in ulcerative colitis-related neoplasms [24]. However, there are no reports of gastric cancer complications being more common in patients with ulcerative colitis or of p53 mutations being more likely to occur in the gastric mucosa, and no association has been demonstrated [25]. Furthermore, this case showed that MUC5AC and MUC6 were positive, and MUC2 was partially positive in the cancerous area, indicating the development of a gastrointestinal phenotype. Although the expression of the gastric phenotype has been reported in high-grade dysplasia or adenocarcinoma occurring with sporadic FGP in HP-uninfected patients [16–18], the expression of the gastrointestinal phenotypes has been reported at a certain frequency in HP-uninfected gastric cancers [26, 27]. The gastrointestinal types are considered to be derived from various cell types and are presumed to be the result of multifactorial carcinogenesis. Based on these results, the possibility of multifactorial carcinogenesis was also suspected in gastric cancers occurring in sporadic FGP.

Gastric adenocarcinoma of fundic-gland type (GA-FG) has been reported to be the most common type of HP-uninfected gastric cancer [28]. GA-FGs occur in the upper part of the stomach and are identified by endoscopic findings as having an elevated shape, especially a submucosal tumor (SMT)-like shape, or a flat or depressed shape [29]. GA-FGs are known to infiltrate the submucosa more frequently among HP-uninfected gastric neoplasms. GA-FGs are immunohistochemically positive for pepsinogen-I, H + /K + ATPase and MUC6 staining. A subtype of GA-FG, gastric adenocarcinoma of fundic-gland mucosa type (GA-FGM), has also been proposed, which differs from GA-FG by being positive for MUC5AC [29]. In the present case, MUC5AC and MUC6 were positive, but pepsinogen I and H + /K + ATPase were negative, showing a different immunostaining attitude and not meeting the definition of GA-FGM. Furthermore, the endoscopic findings were not the SMT-like shape characteristic of GA-FGM, but a well-defined borderline elevated lesion.

In summary, we experienced a case of gastric invasive adenocarcinoma occurring in sporadic FGP without an HP infection. The unusual endoscopic findings of rapid enlargement and reddish tone pushed us to perform endoscopic treatment, which resulted in a correct diagnosis and appropriate treatment.
